# Loss of PKA regulatory subunit 1α aggravates cardiomyocyte necrosis and myocardial ischemia/reperfusion injury

**DOI:** 10.1016/j.jbc.2021.100850

**Published:** 2021-06-01

**Authors:** Yuening Liu, Jingrui Chen, Peng Xia, Constantine A. Stratakis, Zhaokang Cheng

**Affiliations:** 1 Department of Pharmaceutical Sciences, Washington State University, Spokane, Washington, USA; 2 Section on Endocrinology and Genetics, Eunice Kennedy Shriver National Institute of Child Health and Human Development, National Institutes of Health, Bethesda, Maryland, USA

**Keywords:** myocardial infarction, oxidative stress, necrosis, PKA, catecholamine, adrenergic receptor, adenylyl cyclase, mitochondrial permeability transition, cardiomyopathy, Carney complex, AMCMs, adult mouse cardiomyocytes, NRCMs, neonatal rat cardiomyocytes, R1α, PKA regulatory subunit 1α

## Abstract

Reperfusion therapy, the standard treatment for acute myocardial infarction, can trigger necrotic death of cardiomyocytes and provoke ischemia/reperfusion (I/R) injury. However, signaling pathways that regulate cardiomyocyte necrosis remain largely unknown. Our recent genome-wide RNAi screen has identified a potential necrosis suppressor gene *PRKAR1A*, which encodes PKA regulatory subunit 1α (R1α). R1α is primarily known for regulating PKA activity by sequestering PKA catalytic subunits in the absence of cAMP. Here, we showed that depletion of R1α augmented cardiomyocyte necrosis *in vitro* and *in vivo*, resulting in exaggerated myocardial I/R injury and contractile dysfunction. Mechanistically, R1α loss downregulated the Nrf2 antioxidant transcription factor and aggravated oxidative stress following I/R. Degradation of the endogenous Nrf2 inhibitor Keap1 through p62-dependent selective autophagy was blocked by R1α depletion. Phosphorylation of p62 at Ser349 by mammalian target of rapamycin complex 1 (mTORC1), a critical step in p62-Keap1 interaction, was induced by I/R, but diminished by R1α loss. Activation of PKA by forskolin or isoproterenol almost completely abolished hydrogen-peroxide-induced p62 phosphorylation. In conclusion, R1α loss induces unrestrained PKA activation and impairs the mTORC1-p62-Keap1-Nrf2 antioxidant defense system, leading to aggravated oxidative stress, necrosis, and myocardial I/R injury. Our findings uncover a novel role of PKA in oxidative stress and necrosis, which may be exploited to develop new cardioprotective therapies.

Despite the advances in treatment strategies, acute myocardial infarction (MI) remains a major cause of death worldwide (1). MI is characterized by myocardial ischemia owing to coronary artery occlusion. Restoration of blood flow by timely reperfusion can salvage ischemic myocardium and has been widely adopted in clinical MI treatment. Unfortunately, reperfusion therapy itself is associated with excessive reactive oxygen species (ROS) production, which provokes cardiac cell death and contributes to the extent of MI (2). As a major form of cell death in ischemia/reperfusion (I/R) injury, necrosis has great potential to be targeted in MI therapies. However, pharmacologic necrosis inhibitors have rarely progressed to clinical trials, and the translation into better outcomes in patients has been disappointing (3). The poor clinical translation of cardioprotective therapies might be due to our incomplete understanding of the signaling pathways underlying regulated necrosis (4).

To search for novel necrosis regulators, we recently performed a genome-wide RNAi screen and showed that *PRKAR1A* is a highly scored necrosis inhibitory gene (5). *PRKAR1A* encodes the protein kinase A (PKA) regulatory subunit 1α (R1α), which is constitutively expressed in all cell types including cardiomyocytes. In the absence of the second messenger 3′,5′-cyclic adenosine monophosphate (cAMP), R1α sequesters the PKA catalytic subunits and restrains kinase activity. Upon binding with cAMP, R1α undergoes conformational change, resulting in release of the PKA catalytic subunit and subsequent kinase activation. The canonical PKA pathway is initiated by stimulation with catecholamines including epinephrine, norepinephrine, and isoproterenol (6). Catecholamines bind β-adrenergic receptor (β-AR) to induce G_s_-dependent activation of adenylyl cyclase (AC), resulting in cAMP synthesis and PKA activation. Activation of PKA increases cardiac contractility and heart rate through phosphorylating Ca^2+^-handling proteins (6). However, it remains elusive whether PKA modulates necrosis.

In response to oxidative and electrophilic stresses, the body launches the antioxidant defense system mediated by nuclear factor erythroid 2-related factor 2 (Nrf2) (7). The Nrf2 transcription factor binds the antioxidant response element (ARE) to induce expression of antioxidant and cytoprotective genes. Under basal conditions, Nrf2 is rapidly degraded through the ubiquitin-proteasome pathway following polyubiquitination mediated by kelch-like ECH-associated protein 1 (Keap1). In the presence of ROS or electrophiles, Keap1 is oxidized at the cysteine residues, leading to p62-dependent Keap1 degradation and Nrf2 stabilization (8). In the present study, we show that disruption of R1α suppresses Keap1 degradation and impairs Nrf2-mediated antioxidant response. Mechanistically, R1α depletion inhibits mammalian target of rapamycin complex 1 (mTORC1)-mediated p62 phosphorylation at Ser349, resulting in diminished p62-Keap1 interaction and degradation. Following myocardial I/R challenge, R1α loss aggravates oxidative stress and cardiomyocyte necrosis, leading to exaggerated cardiac dysfunction. Our findings suggest that PKA plays a critical role in the regulation of oxidative stress and cardiomyocyte necrosis.

## Results

### Depletion of Prkar1a gene exacerbated cardiomyocyte necrosis *in vitro*

**Figure 1:**
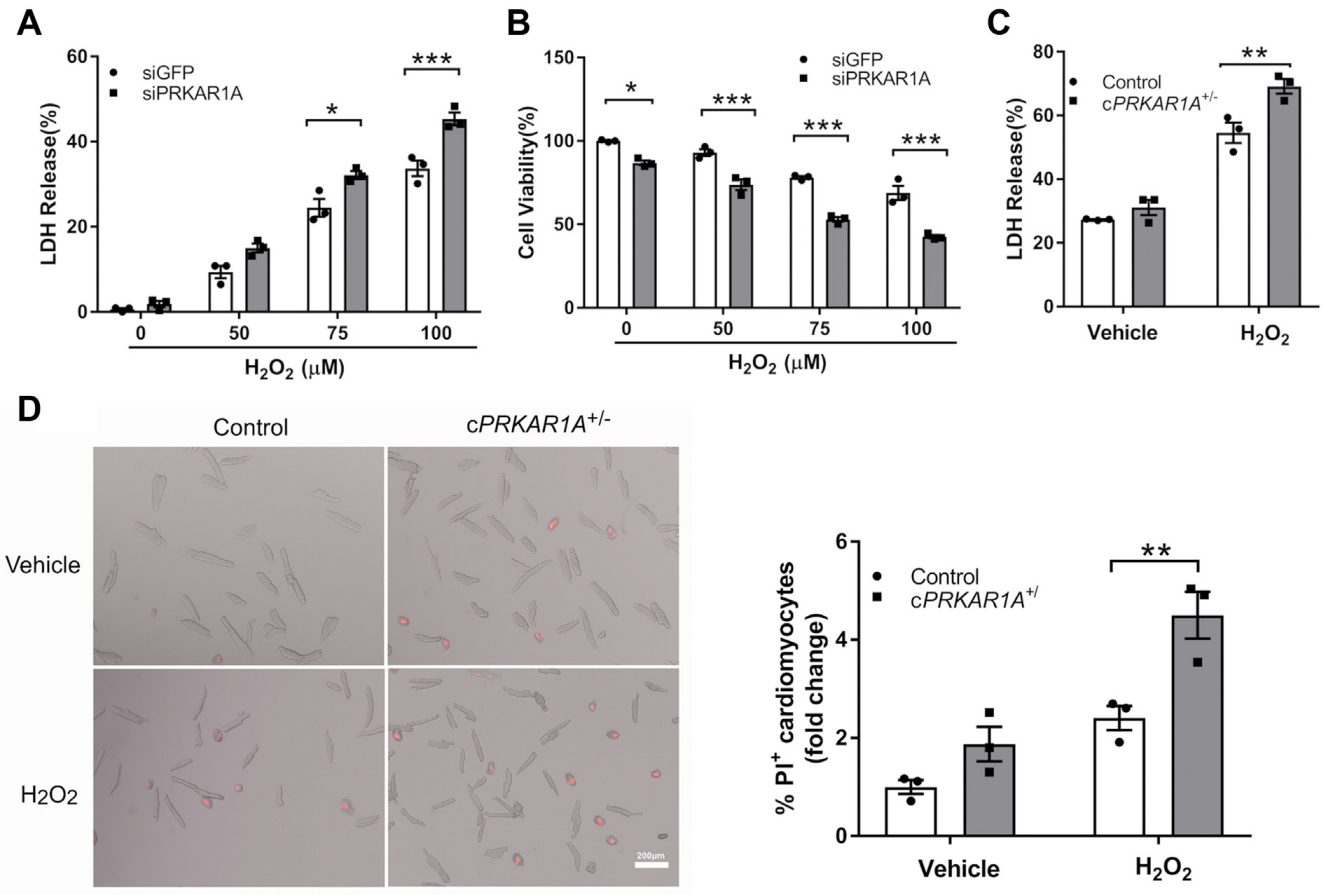
**Depletion of *Prkar1a* gene exacerbated cardiomyocyte necrosis *in vitro*.***A* and *B*, neonatal rat cardiomyocytes (NRCMs) were transfected with control (siGFP) or *Prkar1a* siRNA (siPRKAR1A) for 48 h prior to treatment with various concentrations of H_2_O_2_ for 4 h. *A*, necrosis of NRCMs was determined by LDH release assay (n = 3). Results are mean ± SEM and analyzed using two-way ANOVA with Sidak test. ∗*p* < 0.05, ∗∗∗*p* < 0.001. *B*, viability of NRCMs was determined by MTT assay (n = 3). Two-way ANOVA with Sidak test. ∗*p* < 0.05, ∗∗∗*p* < 0.001. *C* and *D*, adult mouse cardiomyocytes (AMCMs) isolated from control or *cPRKAR1A*^+/−^ mice were treated with H_2_O_2_ (10 μM) for 1 h. *C*, necrosis of AMCMs was determined by LDH release assay (n = 3). Two-way ANOVA with Sidak test. ∗∗*p* < 0.01. *D*, necrosis of AMCMs was determined by PI exclusion assay (n = 3). Scale bar = 200 μm. Two-way ANOVA with Sidak test. ∗∗ *p* < 0.01.

Based on unbiased genome-wide RNAi screening, we previously reported that the *PRKAR1A* gene was a potential necrosis suppressor (5). To validate this finding, neonatal rat cardiomyocytes (NRCMs) were transfected with *Prkar1a* siRNAs prior to incubation with increasing concentrations of H_2_O_2_. Loss of plasma membrane integrity, a hallmark of necrosis, was assessed by lactate dehydrogenase (LDH) release. Treatment with H_2_O_2_ (50–100 μM) for up to 4 h increased LDH release in a dose-dependent manner (Fig. 1*A*). Unlike the apoptosis-inducing agent doxorubicin, H_2_O_2_ did not increase the level of cleaved PARP (Fig. S1, *A* and *B*), a widely used marker of apoptosis. These results suggested that stimulation with H_2_O_2_ in our experimental conditions induced necrosis but not apoptosis in NRCMs. Knockdown of the R1α-encoding gene *Prkar1a* further augmented H_2_O_2_-induced LDH release (Fig. 1*A*) without enhancing PARP cleavage (Fig. S1, *A* and *B*), suggesting that disruption of R1α exacerbated necrosis but not apoptosis. *Prkar1a* gene silencing also reduced cell viability at baseline and following incubation with H_2_O_2_ (Fig. 1*B*). To further validate the antinecrotic role of R1α, adult mouse cardiomyocytes (AMCMs) were isolated from control or cardiac-specific *Prkar1a* heterozygous deficient (*Prkar1a*^flox/+^/*Mlc2v-Cre*^+/−^, hereafter referred to as c*PRKAR1A*^+/−^) mice (9). Heterozygous mice were used in this experiment because homozygous *Prkar1a* knockout mice are embryonically lethal (10, 11, 12, 13). Again, deletion of *Prkar1a* significantly augmented H_2_O_2_-induced LDH release in AMCMs (Fig. 1*C*). *Prkar1a* deficiency also increased propidium iodide (PI) incorporation, another marker of membrane rupture (Fig. 1*D*). Together, these results suggested that depletion of R1α exacerbated oxidative-stress-induced cardiomyocyte necrosis *in vitro*.

To determine whether R1α also antagonizes calcium-overload-induced necrosis, NRCMs transfected with *Prkar1a* siRNAs were challenged with the Ca^2+^ ionophore ionomycin. Knockdown of *Prkar1a* significantly increased ionomycin-induced LDH release (Fig. S2*A*) and reduced cell viability (Fig. S2*B*). The increase in LDH release resulting from *Prkar1a* silencing was attenuated by treatment with the PKA inhibitor PKI, suggesting PKA activation was necessary for necrosis (Fig. S2*C*). In line with these findings, depletion of *Prkar1a* in H9c2 cardiac myoblasts also augmented ionomycin-induced LDH release (Fig. S2*D*) and decreased cell viability (Fig. S2*E*).

### Prkar1a deficiency aggravated myocyte necrosis and myocardial ischemia/reperfusion injury

**Figure 2:**
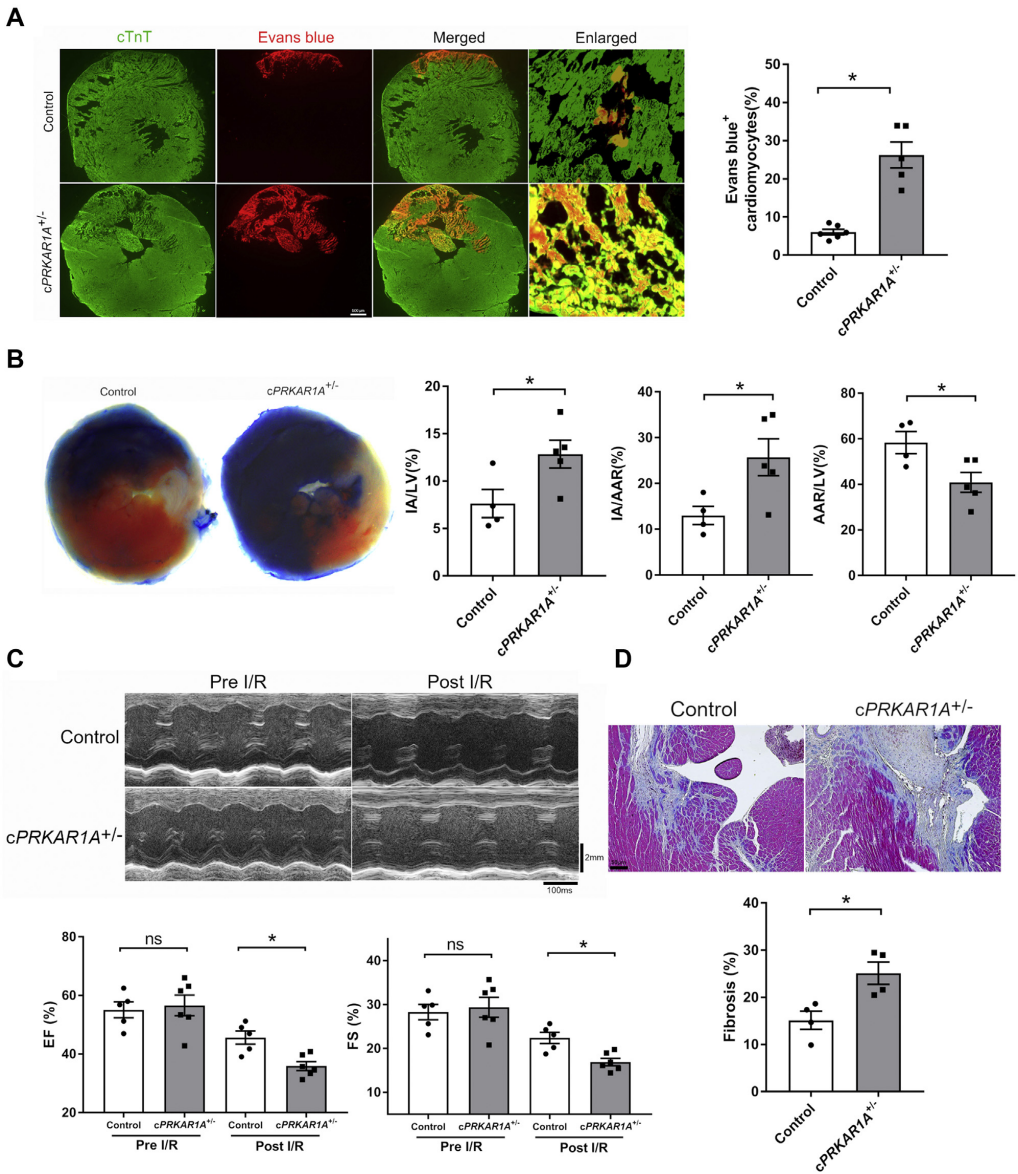
***Prkar1a* deficiency aggravated myocyte necrosis and myocardial ischemia/reperfusion injury.** Littermate control and c*PRKAR1A*^+/−^ mice at 3–4 months of age were subjected to 30 min of ischemia followed by reperfusion. *A*, cardiomyocyte necrosis at 24 h post I/R was assessed by Evans blue dye (*red*) uptake assay. Cardiac troponin T (cTnT, *green*) served as a cardiomyocyte marker. Scale bar = 500 μm. Control, n = 6; c*PRKAR1A*^+/−^, n = 5. Results are mean ± SEM and analyzed using two-tailed Student's *t* test. ∗ *p* < 0.05. *B*, infarct size at 24 h post I/R was examined by Evans blue/TTC staining. Infarct size was calculated as the percentage of infarct area (IA, *white*) to area at risk (AAR, *white* and *red*) or left ventricle (LV, *white*, *red* and *blue*). Control, n = 4; c*PRKAR1A*^+/−^, n = 5. Two-tailed Student's *t* test. ∗ *p* < 0.05. *C*, left ventricular systolic function was evaluated by echocardiography before I/R and 1 week post I/R. Control, n = 5; c*PRKAR1A*^+/−^, n = 6. Two-way ANOVA with Sidak test. ∗ *p* < 0.05. *D*, cardiac fibrosis was evaluated at 8 weeks post I/R *via* Masson's Trichrome staining. Scale bar = 50 μm. Fibrosis was defined as the percentage of fibrotic area (*blue*) to left ventricle. Control, n = 4; c*PRKAR1A*^+/−^, n = 4. Two-tailed Student's *t* test. ∗ *p* < 0.05. EF, ejection fraction; FS, fractional shortening; ns, not significant.

Necrosis is a major form of cell death in MI with reperfusion (4). To test the hypothesis that *Prkar1a* deficiency exaggerates necrosis *in vivo*, c*PRKAR1A*^+/−^ or control mice were subjected to 30 min of ischemia followed by 24 h of reperfusion. Necrotic cardiomyocytes were delineated with Evans blue, a red fluorescent dye for the assessment of sarcolemmal integrity *in vivo* (14). Evans blue signal was not detectable in normal hearts from either genotype (data not shown), indicating the absence of necrosis at baseline. By contrast, Evans-blue-positive cardiomyocytes were clearly visible post I/R and were significantly increased by *Prkar1a* ablation (Fig. 2*A*). *Prkar1a* deficiency also increased infarct size as measured by the percentage of infarct area to area at risk or the total left ventricle (Fig. 2*B*). Interestingly, we repeatedly observed a smaller area at risk in c*PRKAR1A*^+/−^ hearts (Fig. 2*B*), possibly due to exaggerated penetration of Evans blue through the permeabilized sarcolemma. In agreement with our previous study (9), basal heart function was comparable between both groups of mice (Fig. 2*C*). At 1 week post I/R, however, left ventricular ejection fraction (EF) and fractional shortening (FS) were significantly lower in c*PRKAR1A*^+/−^ than in control mice (Fig. 2*C*). At baseline, myocardial fibrosis was minimal and comparable in both genotypes (Fig. S3). Notably, fibrotic area was significantly larger in c*PRKAR1A*^+/−^ hearts than in controls at 8 weeks post I/R (Fig. 2*D*). Collectively, *Prkar1a* deficiency exacerbated cardiomyocyte necrosis following I/R, leading to increased infarct size, reduced cardiac function, and aggravated fibrotic remodeling.

### Disruption of R1α augmented oxidative stress in cardiomyocytes *in vivo* and *in vitro*

**Figure 3:**
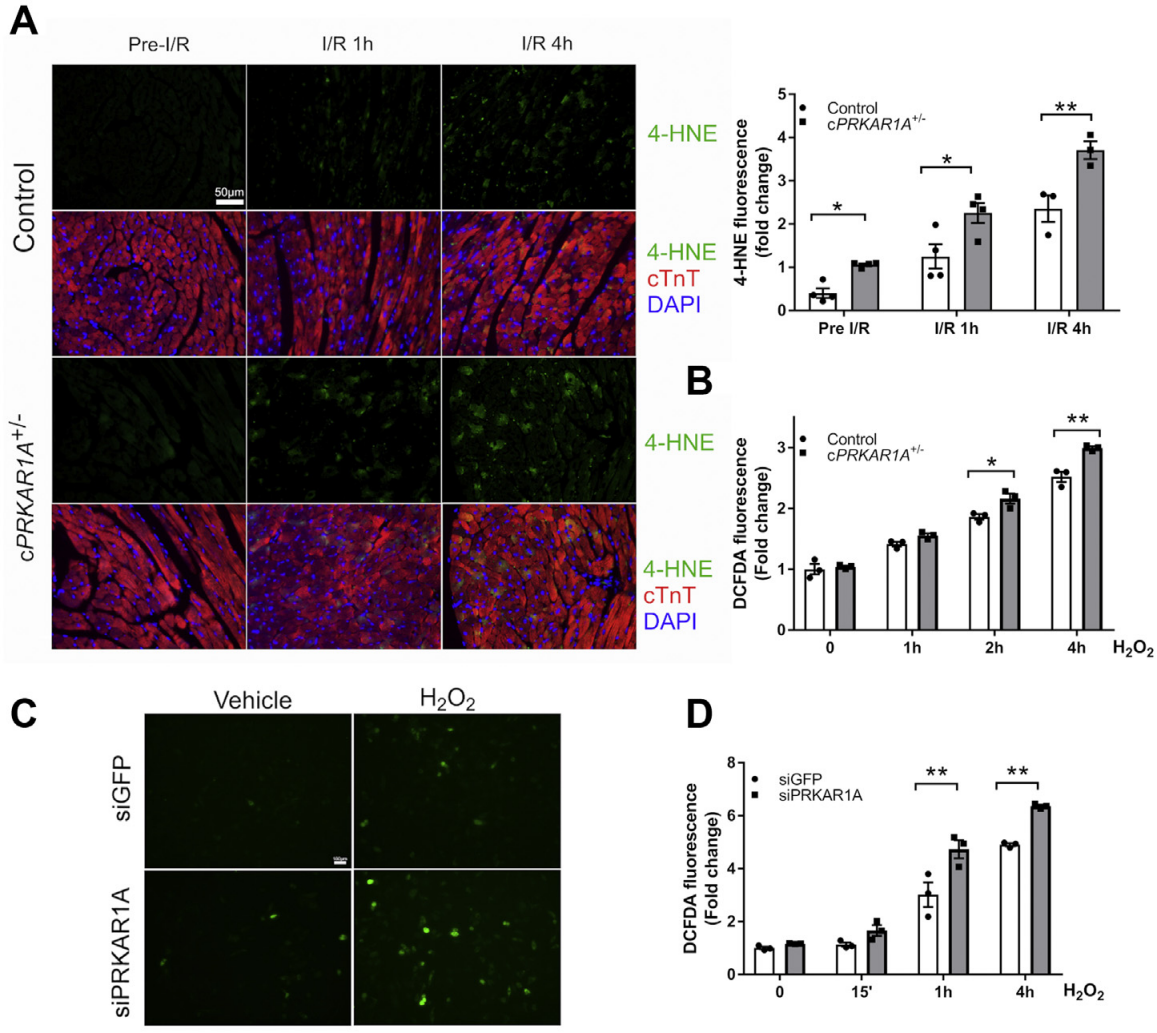
**Disruption of R1α augmented oxidative stress in cardiomyocytes *in vivo* and *in vitro*.***A*, control and c*PRKAR1A*^+/−^ hearts were harvested before, 1 h or 4 h post I/R (n = 3–4 per group each time point). Heart sections were stained for the lipid peroxidation marker 4-hydroxynoneal (4-HNE, *green*), cTnT (*red*), and nuclei (DAPI, *blue*). Scale bar = 50 μm. Results are mean ± SEM and analyzed using two-way ANOVA with Sidak test. ∗ *p* < 0.05, ∗∗ *p* < 0.01. *B*, AMCMs isolated from control and c*PRKAR1A*^+/−^ hearts were treated with H_2_O_2_ (10 μM) for 1 h (n = 3). Oxidative stress was assessed by staining with H2DCFDA. Fluorescence intensity of H2DCFDA was quantified using a fluorescence plate reader. Two-way ANOVA with Sidak test. ∗ *p* < 0.05, ∗∗ *p* < 0.01. *C* and *D*, NRCMs transfected with siGFP or siPRKAR1A were treated with H_2_O_2_ (100 μM) for 1 h and then stained with H2DCFDA. *C*, representative images of H2DCFDA fluorescence under a fluorescent microscope. Scale bar = 100 μm. *D*, fluorescence intensity of H2DCFDA was quantified using a fluorescence plate reader (n = 3). Two-way ANOVA with Sidak test. ∗∗ *p* < 0.01.

Since reperfusion-induced ROS generation is a major cause of necrotic cell death (2), we examined whether both genotypes were under similar levels of oxidative stress following I/R challenge. In control hearts, I/R rapidly increased the fluorescence intensity of 4-hydroxynoneal (4-HNE), a product of lipid peroxidation and a widely used biomarker for oxidative stress (Fig. 3*A*). When compared with controls, c*PRKAR1A*^+/−^ hearts displayed higher levels of 4-HNE both in basal conditions and after I/R. To determine whether *Prkar1a* deficiency aggravates oxidative stress in a cardiomyocyte autonomous fashion, AMCMs were isolated from control or c*PRKAR1A*^+/−^ hearts prior to incubation with H_2_O_2_. As shown in Figure 3*B*, ablation of *Prkar1a* significantly augmented H_2_O_2_-induced 2ʹ,7ʹ-dichlorodihydrofluorescin diacetate (H2DCFDA) fluorescence, an indicator of overall oxidative stress. Moreover, silencing of *Prkar1a* in NRCMs also increased H2DCFDA fluorescence following H_2_O_2_ stimulation (Fig. 3, *C* and *D*). Therefore, disruption of R1α augmented oxidative stress in cardiomyocytes.

### Lack of R1α downregulated the antioxidant transcription factor Nrf2 during oxidative stress

**Figure 4:**
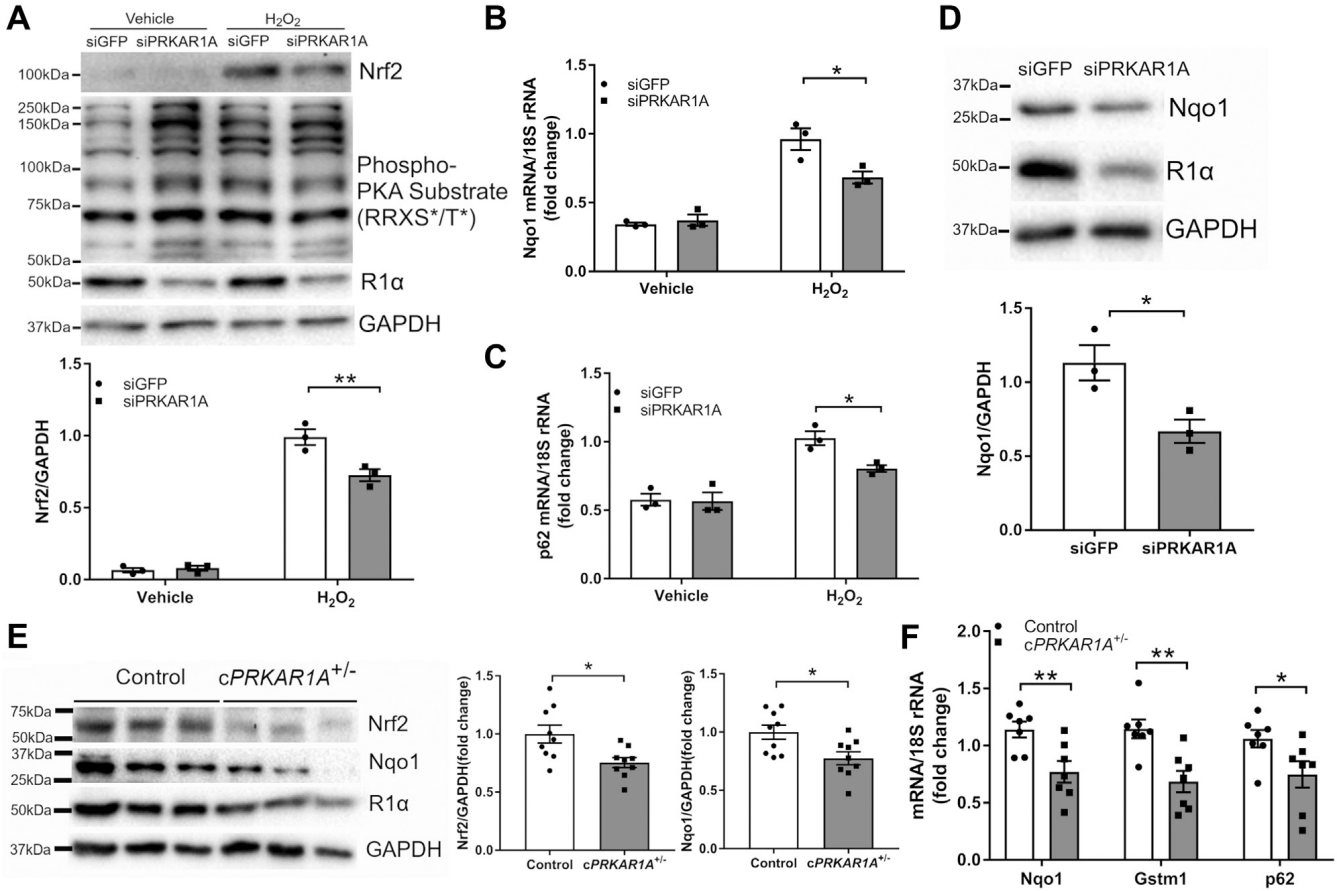
**Lack of R1α downregulated the antioxidant transcription factor Nrf2 during oxidative stress.***A*, NRCMs were transfected with control (siGFP) or *Prkar1a* siRNA (siPRKAR1A) for 48 h prior to incubation with H_2_O_2_ (100 μM) for 1 h (n = 3). Protein levels were analyzed by western blotting. Results are mean ± SEM and analyzed using two-way ANOVA with Sidak test. ∗∗ *p* < 0.01. *B–D*, NRCMs transfected with siGFP or siPRKAR1A were treated with H_2_O_2_ (100 μM) for 4 h. *B* and *C*, quantitative RT-PCR analysis of mRNA levels (n = 3). Two-way ANOVA with Sidak test. ∗ *p* < 0.05. *D*, western blot analysis of protein levels (n = 3). Two-way ANOVA with Sidak test. ∗ *p* < 0.05. *E* and *F*, control and c*PRKAR1A*^+/−^ hearts were harvested at 1 h post I/R. *E*, western blot analysis of protein levels (n = 9 per group). Two-tailed Student's *t* test. ∗ *p* < 0.05. *F*, quantitative RT-PCR analysis of mRNA levels (n = 7 per group). Two-tailed Student's *t* test. ∗ *p* < 0.05.

Intracellular redox homeostasis is maintained by a variety of antioxidant defense systems including the Nrf2 pathway (7). Under basal conditions, Nrf2 was expressed at low level in cardiomyocytes (Fig. 4*A*). Stimulation with H_2_O_2_ dramatically increased Nrf2 protein level as an adaptive response to protect against oxidative damage. Intriguingly, lack of R1α provoked PKA kinase activation at baseline (as determined by the levels of phosphorylated PKA substrates) and blunted H_2_O_2_-induced upregulation of Nrf2 (Fig. 4*A*). In addition, H_2_O_2_ treatment increased the mRNA levels of established Nrf2 transcriptional targets Nqo1 and p62, which were diminished by *Prkar1a* depletion (Fig. 4, *B* and *C*). Lack of R1α also reduced Nqo1 protein level after H_2_O_2_ challenge (Fig. 4*D*). To further validate these findings *in vivo*, control and c*PRKAR1A*^+/−^ mice were subjected to I/R and hearts were collected at 1 h. Deficit of R1α again reduced Nrf2 and Nqo1 protein levels in mouse heart (Fig. 4*E*). Interestingly, Nrf2 was detected at ∼100 kDa in NRCMs (Fig. 4*A*) and ∼67 kDa in mouse heart (Fig. 4*E*). When compared with controls, c*PRKAR1A*^+/−^ hearts exhibited lower mRNA levels of Nrf2 targets Nqo1, Gstm1, and p62 (Fig. 4*F*). These results suggested that lack of R1α repressed the antioxidant transcription factor Nrf2 and thus impaired the endogenous defense response against oxidative stress.

### Activation of PKA increased Keap1 and p62 protein stability without inhibiting general autophagy

**Figure 5:**
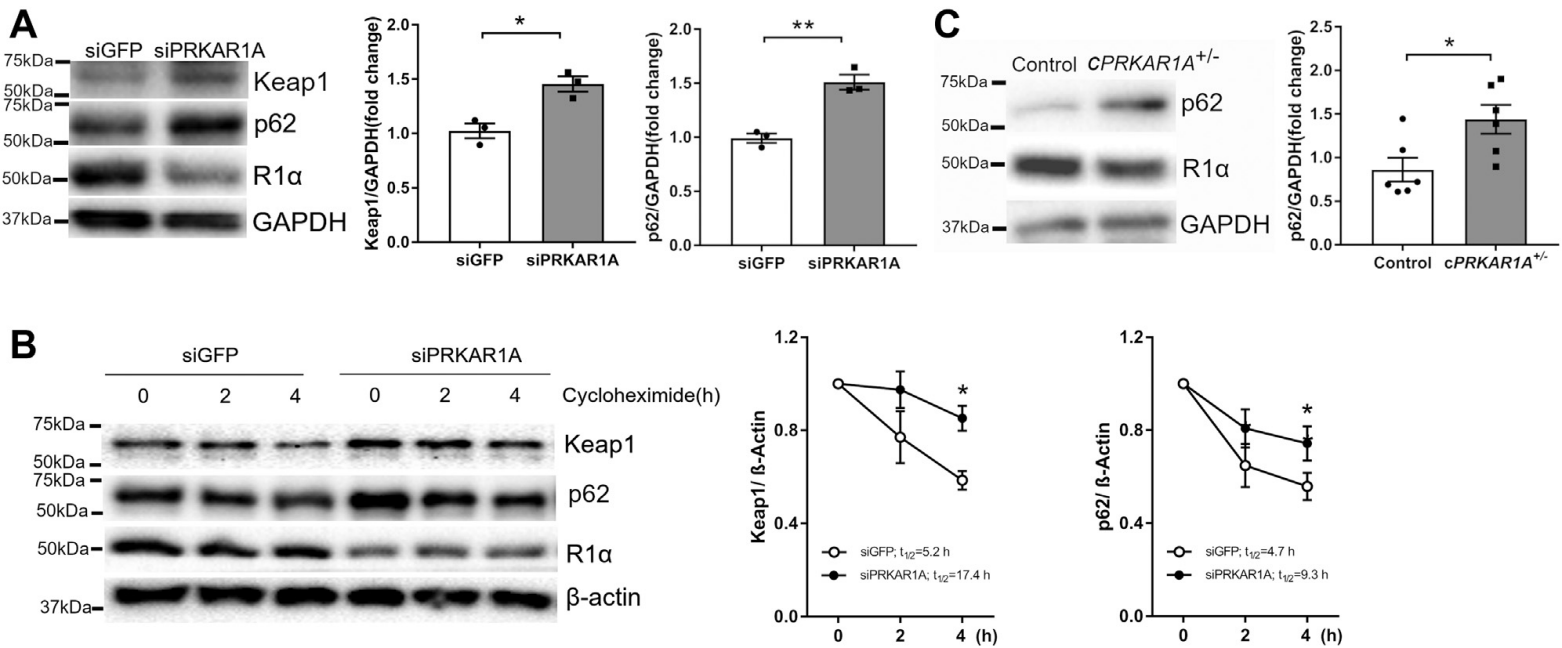
**Activation of PKA increased Keap1 and p62 protein stability.***A*, NRCMs were transfected with control (siGFP) or *Prkar1a* siRNA (siPRKAR1A) for 48 h (n = 3). Protein levels were analyzed by western blotting. Results are mean ± SEM and analyzed using two-tailed Student's *t* test. ∗ *p* < 0.05, ∗∗ *p* < 0.01. *B*, NRCMs transfected with siGFP or siPRKAR1A were incubated with the protein synthesis inhibitor cycloheximide (10 μg/ml) for various periods of time (n = 3). Western blotting revealed that knockdown of *Prkar1a* increased Keap1 and p62 protein stability. Two-way ANOVA with Sidak test. ∗ *p* < 0.05 *versus* siGFP. *C*, western blot analysis of protein levels in normal control and c*PRKAR1A*^+/−^ mice hearts (n = 6 per group). Two-tailed Student's *t* test. ∗ *p* < 0.05.

In nonstressed conditions, Nrf2 undergoes rapid proteasomal degradation that is dependent on Keap1 (7). R1α deficit-related Nrf2 repression during oxidative stress (Fig. 4) was associated with increased Keap1 protein at baseline (Fig. 5*A*). Incubation with the protein synthesis inhibitor cycloheximide revealed that lack of R1α dramatically elongated the half-life of Keap1 protein from 5.2 h to 17.4 h (Fig. 5*B*), indicating increased protein stability. Since Keap1 is mainly degraded through autophagy (8), stabilization of Keap1 could be caused by a defect in autophagy. Indeed, disruption of R1α also increased the protein level and stability of p62, a well-known autophagy substrate and a reporter of autophagic activity (Fig. 5, *A* and *B*). Since *Prkar1a* depletion did not increase p62 mRNA level (Fig. 4*C*), the upregulation of p62 protein was primarily mediated by increased p62 protein stability. Surprisingly, lack of R1α failed to reduce the level of lipidated microtubule-associated protein light chain 3 (LC3-II), a widely used autophagosome marker (Fig. S4*A*). The rate of LC3-II turnover, evaluated with the lysosomal inhibitor chloroquine as described previously (15), was comparable between control and *Prkar1a*-depleted cells (Fig. S4*B*), suggesting that knockdown of R1α did not inhibit autophagic flux. Therefore, lack of R1α did not block the nonselective general autophagy, but specifically inhibited autophagic degradation of Keap1 and p62. To further determine whether the effects of R1α loss on autophagy are recapitulated by pharmacological PKA activation, NRCMs were incubated with the adenylyl cyclase agonist forskolin or the β-AR agonist isoproterenol. Both forskolin and isoproterenol dramatically increased p62 level in a dose-dependent manner, without reducing LC3-II level (Fig. S4*C*). Moreover, treatment with forskolin or isoproterenol did not inhibit LC3-II turnover, indicating unaltered autophagic flux (Fig. S4*D*). Conversely, treatment with the PKA inhibitor H89 markedly accelerated p62 degradation (Fig. S5), suggesting that PKA activation stabilizes p62. Importantly, loss of R1α also increased p62 protein level in mouse heart (Fig. 5*C*).

### ROS induced p62 phosphorylation at Ser349, leading to cardiac Keap1-Nrf2 activation

**Figure 6:**
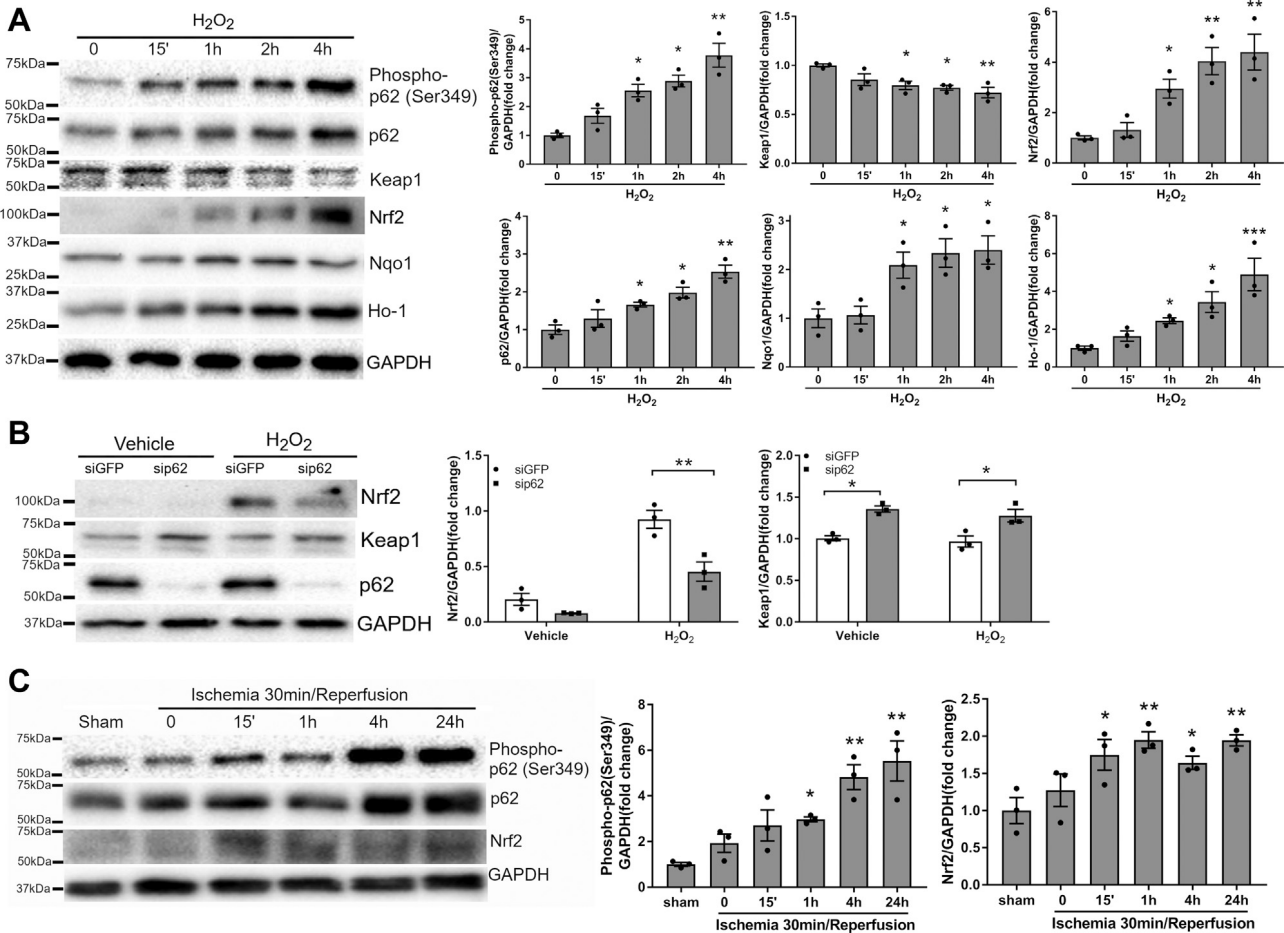
**ROS induced p62 phosphorylation at Ser349, leading to cardiac Keap1-Nrf2 activation.***A*, NRCMs were treated with H_2_O_2_ (100 μM) for various periods of time (n = 3). Protein levels were analyzed by western blotting. Results are mean ± SEM and analyzed using one-way ANOVA with Dunnett's test. ∗ *p* < 0.05, ∗∗ *p* < 0.01, ∗∗∗ *p* < 0.001 *versus* time 0. *B*, NRCMs were transfected with control (siGFP) or p62 siRNA (sip62) for 48 h prior to incubation with H_2_O_2_ (100 μM) for 1 h (n = 3). Protein levels were analyzed by western blotting. Two-way ANOVA with Sidak test. ∗ *p* < 0.05, ∗∗ *p* < 0.01. *C*, C57BL/6 mice were subjected to 30 min of ischemia followed by reperfusion for various periods of time (n = 3 per time point). Heart lysates were subjected to western blotting. One-way ANOVA with Dunnett's test. ∗ *p* < 0.05, ∗∗ *p* < 0.01 *versus* Sham.

As an autophagy adaptor, p62 recruits a selective group of substrates to autophagosomes for degradation. For example, Keap1 degradation occurs through p62-dependent selective autophagy (8). The binding affinity of p62 for Keap1 is increased by p62 phosphorylation at Ser349 (16). However, it remains unknown whether cardiac ROS stimulates p62 phosphorylation to trigger Keap1 degradation and Nrf2 activation. As shown in Figure 6*A*, treatment with H_2_O_2_ induced p62 phosphorylation at Ser349 in a time-dependent manner. Phosphorylation of p62 was accompanied by a decline in Keap1 level, as well as upregulation of Nrf2 and its transcriptional targets p62, Nqo1 and Ho-1 (Fig. 6*A*). These data were in agreement with previous findings that p62 phosphorylation played a causal role in Keap1 degradation and Nrf2 activation (16). Similar to R1α (Fig. 4*A*), knockdown of p62 increased Keap1 level and markedly diminished H_2_O_2_-induced Nrf2 upregulation (Fig. 6*B*), suggesting that p62 is necessary for Nrf2 activation. Moreover, myocardial I/R time-dependently induced p62 phosphorylation at Ser349, which was also associated with Nrf2 upregulation (Fig. 6*C*). Together, these results suggested that phosphorylation of p62 at Ser349 was a key cardiac defense mechanism against oxidative stress.

### Phosphorylation of p62 at Ser349 was diminished by genetic or pharmacologic PKA activation

**Figure 7:**
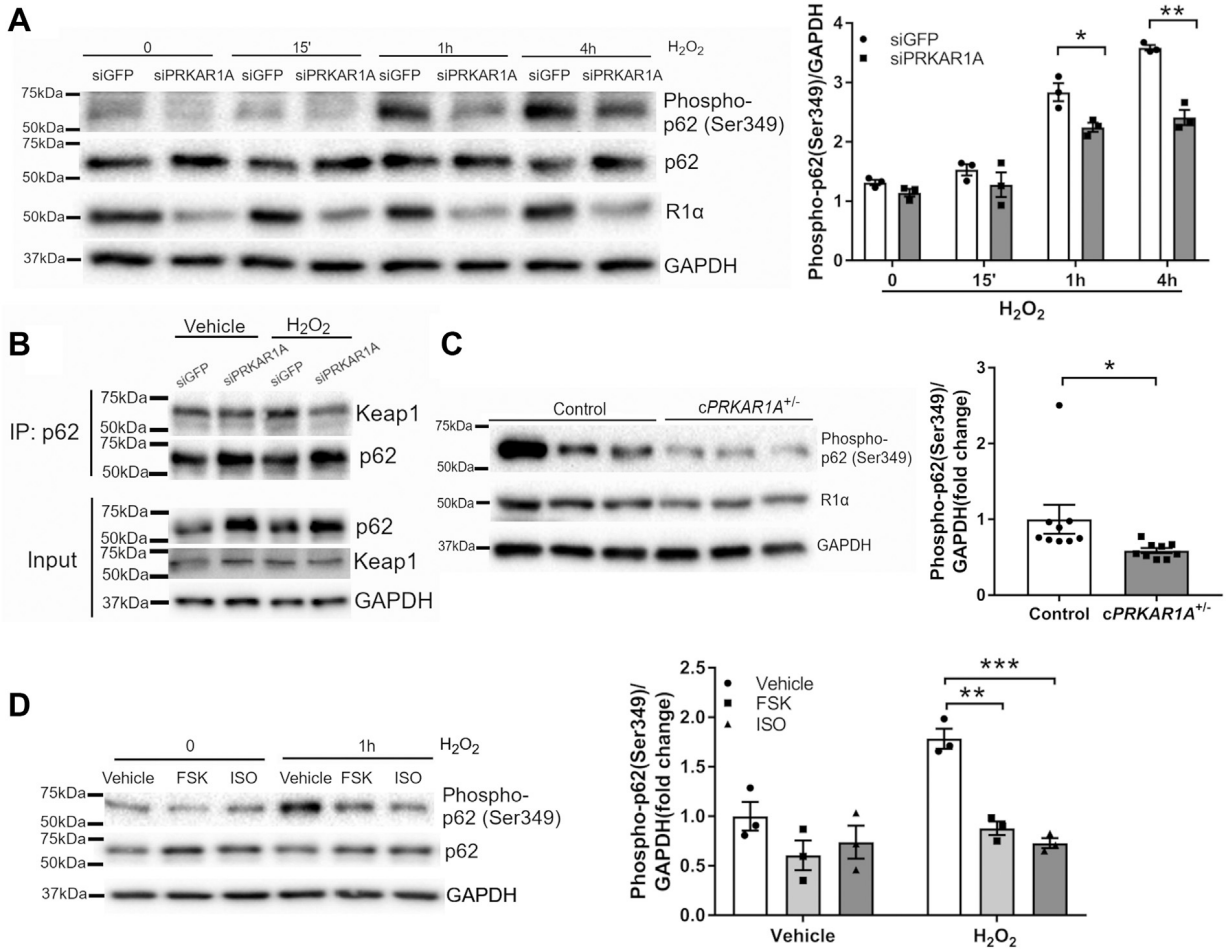
**Phosphorylation of p62 at Ser349 was diminished by genetic or pharmacologic PKA activation.***A*, NRCMs were transfected with control (siGFP) or *Prkar1a* siRNA (siPRKAR1A) for 48 h prior to incubation with H_2_O_2_ (100 μM) for various periods of time (n = 3). Protein levels were analyzed by western blotting. Results are mean ± SEM and analyzed using two-way ANOVA with Sidak test. ∗ *p* < 0.05, ∗∗ *p* < 0.01. *B*, NRCMs were transfected with siGFP or siPRKAR1A for 48 h prior to incubation with H_2_O_2_ (100 μM) for 1 h. Protein lysates were immunoprecipitated (IP) with anti-p62 antibody followed by western blotting with the indicated antibodies. Image represents three independent experiments. *C*, control and c*PRKAR1A*^+/−^ mice were subjected to 30 min of ischemia followed by 1 h of reperfusion (n = 9 per group). Protein levels in heart lysates were analyzed by western blotting. Two-tailed Student's *t* test. ∗ *p* < 0.05. *D*, NRCMs were pretreated with forskolin (FSK, 10 μM) or isoproterenol (ISO, 10 μM) for 4 h prior to incubation with H_2_O_2_ (100 μM) for 1 h (n = 3). Protein levels were analyzed by western blotting. Two-way ANOVA with Sidak test. ∗∗ *p* < 0.01, ∗∗∗ *p* < 0.001.

It is known that p62 competes with Nrf2 to bind Keap1, resulting in Nrf2 stabilization and activation (17). We were intrigued by our findings that R1α loss increased p62 protein but repressed Nrf2. Therefore, we interrogated whether lack of R1α inactivated p62. Indeed, disruption of R1α abrogated H_2_O_2_-induced p62 phosphorylation at Ser349 (Fig. 7*A*) and p62-Keap1 interaction (Fig. 7*B*). These results suggested that loss of R1α compromised the adaptor function of p62, which was necessary for Keap1 degradation by p62-dependent selective autophagy (7). Importantly, c*PRKAR1A*^+/−^ hearts exhibited significantly lower level of phospho-p62 (Ser349) following I/R (Fig. 7*C*). We next examined whether pharmacologic PKA activation also recapitulates the effect of R1α loss. As expected, treatment with forskolin or isoproterenol almost completely abolished H_2_O_2_-induced p62 phosphorylation at Ser349 (Fig. 7*D*). Therefore, activation of PKA suppressed ROS-induced p62 phosphorylation, leading to repression of the Keap1-Nrf2 antioxidant axis.

### Disruption of R1α abolished ROS-induced mTORC1 activation

**Figure 8:**
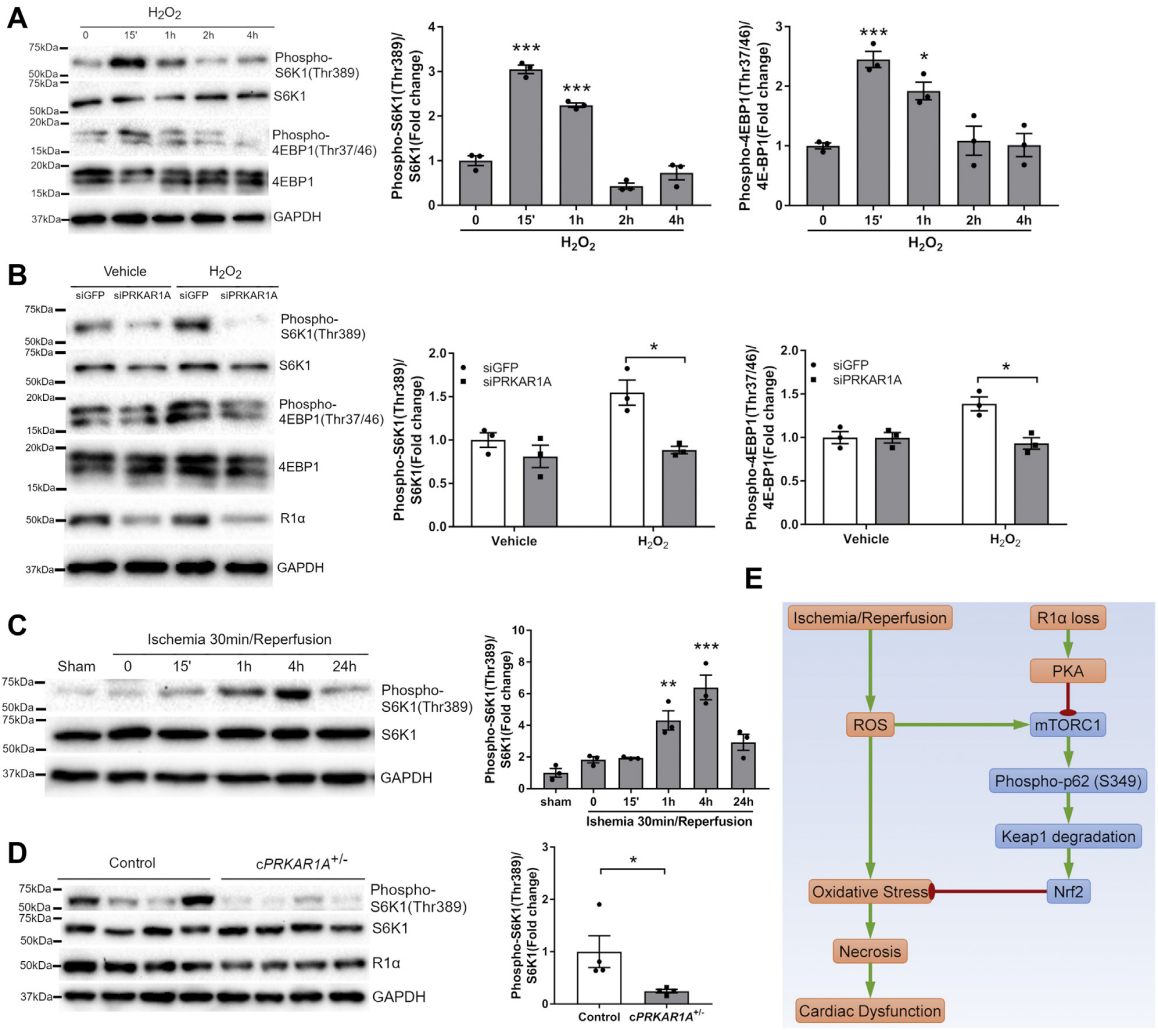
**Disruption of R1α abolished ROS-induced mTORC1 activation.***A*, NRCMs were treated with H_2_O_2_ (100 μM) for various periods of time (n = 3). Protein levels were analyzed by western blotting. Results are mean ± SEM and analyzed using one-way ANOVA with Dunnett's test. ∗ *p* < 0.05, ∗∗∗ *p* < 0.001 *versus* time 0. *B*, NRCMs were transfected with control (siGFP) or *Prkar1a* siRNA (siPRKAR1A) for 48 h prior to incubation with H_2_O_2_ (100 μM) for 1 h (n = 3). Protein levels were analyzed by western blotting. Two-way ANOVA with Sidak test. ∗ *p* < 0.05. *C*, C57BL/6 mice were subjected to 30 min of ischemia followed by reperfusion for various periods of time (n = 3 per time point). Heart lysates were subjected to western blotting. One-way ANOVA with Dunnett's test. ∗∗ *p* < 0.01, ∗∗∗ *p* < 0.001 *versus* Sham. *D*, control and c*PRKAR1A*^+/−^ hearts were harvested at 1 h post I/R (n = 4 per group). Protein levels were analyzed by western blotting. Two-tailed Student's *t* test. ∗ *p* < 0.05. *E*, schematic summary. Ischemia/reperfusion stimulates production of reactive oxygen species (ROS), leading to oxidative-stress-induced necrosis and cardiac dysfunction. On the other hand, ROS also induces mTORC1-mediated phosphorylation of p62 at Ser349, resulting in activation of the Keap1/Nrf2 antioxidant defense system to limit oxidative stress. Unrestrained PKA activation caused by R1α loss suppresses the mTORC1-p62-Keap1-Nrf2 antioxidant response, resulting in aggravated reperfusion injury. *Arrow*, activation; bar-headed line, inhibition.

Since the Ser349 site of p62 is phosphorylated by mTORC1 (16), we next determined whether cardiac ROS exposure induces mTORC1 activation. Treatment of NRCMs with H_2_O_2_ transiently increased the levels of phospho-S6K1 (Thr389) and phospho-4EBP1 (Thr37/46), two well-established mTORC1 substrates (Fig. 8*A*). Intriguingly, knockdown of *Prkar1a* abolished H_2_O_2_-induced phosphorylation of S6K1 and 4EBP1 (Fig. 8*B*). In line with our *in vitro* findings, I/R also induced cardiac S6K1 phosphorylation in a time-dependent manner, indicating mTORC1 activation (Fig. 8*C*). Ablation of *Prkar1a* significantly reduced the level of phospho-S6K1 (Thr389) in mouse heart post I/R (Fig. 8*D*). Collectively, these results suggested that disruption of R1α abrogated ROS-induced mTORC1 activation, thereby impaired the p62-Keap1-Nrf2 antioxidant defense system (Fig. 8*E*).

## Discussion

Necrotic cell death is a key mediator of tissue damage in various ischemic, inflammatory, and degenerative diseases. Studies over the past decades suggest that necrosis can be a highly organized process that is amenable to pharmacological interventions (4). Hence, exploring the necrosis signaling pathways may open new avenues for the development of novel therapies. In the present study, we demonstrated that loss of R1α exacerbated cardiomyocyte necrosis *in vitro* and *in vivo*, leading to aggravated myocardial I/R injury. Mechanistically, disruption of R1α abolished mTORC1-mediated p62 phosphorylation at Ser349, thereby inhibited the endogenous Keap1-Nrf2 antioxidant activity and augmented oxidative stress (Fig. 8*E*). Our results suggest that elevated PKA activity may contribute to myocardial I/R injury through increasing oxidative stress and necrosis. In agreement with our findings, a most recent study reveals that R1α localizes at the lysosome to inhibit Ca^2+^ release and protect against *ex vivo* I/R injury (18).

Catecholamine stimulation induces activation of the AC/cAMP/PKA pathway (6). Interestingly, acute exposure to high level of catecholamine increases sarcolemmal permeability and provokes cardiomyocyte necrosis (19, 20, 21). It is believed that catecholamines induce necrosis *via* triggering intracellular Ca^2+^ overload, owing to PKA-dependent increases in Ca^2+^ influx through L-type Ca^2+^ channel Ca_v_1.2 and SR Ca^2+^ leak through ryanodine receptor 2 (19, 22). However, a direct evidence of the connection between PKA and necrosis is currently lacking. We previously showed that disruption of the PKA regulatory subunit R1α induces constitutive PKA activation (9). In the current study, we demonstrated that loss of R1α suppressed Nrf2 activation following I/R, resulting in aggravated oxidative stress and necrosis. In line with our data, catecholamine exposure induces oxidative stress, which is associated with repression of Nrf2 (23). Moreover, overexpression of AC5, a major cardiac AC isoform, increases oxidative stress by downregulation of the Nrf2 target gene manganese superoxide dismutase (24). Therefore, PKA-mediated repression of the Nrf2 antioxidant response is likely an important cause of myocardial injury. In addition, PKA may also increase ROS production by suppressing cytochrome *c* oxidase (25). Interestingly, ROS-induced R1α dimerization further stimulates PKA activation in cardiomyocytes (26), indicating a vicious cycle between PKA activation and oxidative stress. We have shown that loss of R1α provokes PKA-dependent phosphorylation of the mitochondrial fission protein Drp1 at Ser637, resulting in mitochondrial elongation (9). Intriguingly, enlarged mitochondria are more susceptible to mitochondrial permeability transition pore (mPTP) opening and necrosis (14, 27). Thus PKA-mediated mitochondrial elongation may also predispose cardiomyocytes to necrosis. Together, these studies suggest that PKA activation may contribute to necrosis through calcium overload, oxidative stress, and mitochondrial enlargement.

It is known that Nrf2 protects against I/R injury and necrosis. Following myocardial I/R, Nrf2 knockout mice exhibit increased infarct size and reduced cardiac function compared with controls (28, 29). Nrf2 also mediates the cardioprotective effects of ischemic preconditioning (28), sulforaphane (29), glucocorticoids (30), and fumarate (31). In addition, ablation of Nrf2 aggravates pressure-overload-induced myocardial necrosis (32), whereas overexpression of Nrf2 prevents puromycin-induced necrosis in adult cardiomyocytes (33). Nrf2 activation also mitigates liver necrosis in a mouse model of hepatic I/R or sickle cell disease (34, 35). Interestingly, Nrf2 appears to suppress necrosis without altering apoptosis (33, 34). Since disruption of R1α similarly augmented necrosis but not apoptosis (Fig. S1), repression of Nrf2 is likely a central mechanism underlying the exaggerated I/R injury and necrosis in R1α-deficient heart.

Nrf2 undergoes ubiquitination and proteasomal degradation mediated by Keap1, an E3 ubiquitin ligase adaptor. In contrast, Keap1 is subjected to degradation *via* p62-dependent selective autophagy. As an Nrf2 transcriptional target, p62 mediates Keap1 degradation and Nrf2 activation in a positive feedback loop (7, 36). Interaction between p62 and Keap1, a key step in p62-mediated Keap1 degradation, requires phosphorylation of p62 at Ser349 by mTORC1 (16). In the current study, we showed that disruption of R1α inhibited mTORC1-dependent p62 phosphorylation, leading to suppression of the Keap1-Nrf2 antioxidant axis. Our results are in agreement with recent findings that PKA inhibits mTORC1 activity *via* phosphorylation of the mTORC1 component Raptor at Ser791 (37).

In this study, we showed that ROS exposure induced Nrf2-mediated transcription of p62, resulting in elevated p62 protein level. Intriguingly, R1α loss repressed Nrf2-mediated p62 transcription, but still upregulated p62 protein due to increased p62 protein stability. Degradation of p62 protein is accelerated by Keap1-mediated ubiquitination of p62 at lysine 420 (38). Since R1α loss diminished p62-Keap1 interaction, PKA likely inhibited Keap1-mediated p62 degradation. Although p62 protein is upregulated by both ROS stimulation and PKA activation, the autophagy adaptor function of p62 (as measured by phosphorylation of p62 at Ser349) is enhanced by ROS but repressed by PKA. Therefore, PKA antagonizes ROS-induced activation of the p62-Keap1-Nrf2 antioxidant system.

Our study has important clinical implications. MI with reperfusion triggers extensive necrotic cell death, leading to systolic dysfunction and eventually heart failure (2). Minimizing cell death has been proposed as a promising cardioprotective therapy (3). To date, the only antinecrotic drug evaluated in clinical trial for cardioprotection is cyclosporine A (3), which inhibits mPTP opening. Unfortunately, cyclosporine A fails to improve clinical outcomes of MI in phase III clinical trials (39, 40). Therefore, it has become increasingly necessary to identify additional drug target in the necrosis pathway (41). Our current study reveals that loss of R1α provokes PKA activation and exacerbates cardiomyocyte necrosis following I/R, indicating a pronecrotic role of PKA. Our results suggest that PKA may serve as a novel drug target in the treatment of MI. Indeed, myocardial I/R injury is attenuated by administration of the PKA inhibitors H89 or PKI (25, 42, 43).

In humans, the R1α encoding gene *PRKAR1A* is subject to inactivating mutations or deletions (44). *PRKAR1A* haploinsufficiency is the primary cause of Carney complex (CNC), a hereditary syndrome characterized by pigmented lesions and benign tumors. Based on our findings, we predict that CNC patients are more susceptible to reperfusion injury following MI. Pharmacologic inhibition of PKA could be an effective adjuvant therapy during the treatment of MI particularly in these patients. In addition to necrosis, R1α also regulates cardiomyocyte proliferation and hypertrophy. Global *Prkar1a* null mice are embryonic lethal because unrestrained PKA activation results in a defect in heart tube formation (10, 11). Cardiac-specific homozygous ablation of *Prkar1a* induces left ventricular noncompaction and embryonic lethality, through PKA-dependent suppression of cardiomyocyte proliferation (12, 13). Recently, we demonstrate that cardiac-specific *Prkar1a* deletion suppresses hypertrophic growth during myocardial development without altering cardiac function (9). Collectively, these studies suggest that PKA regulates many aspects of cardiac development and disease.

In conclusion, the current study reveals that unrestrained PKA activation caused by R1α loss aggravates oxidative stress, cardiomyocyte necrosis, and myocardial I/R injury. Mechanistically, disruption of R1α suppresses mTORC1-p62-dependent degradation of Keap1, resulting in repression of Nrf2-mediated antioxidant response. These results uncover a critical role of PKA in the regulation of oxidative stress and necrosis. Our findings suggest that PKA may represent a promising drug target for preventing reperfusion injury and improving clinical outcomes of MI.

## Experimental procedures

### Animals

c*PRKAR1A*^+/−^ mice were generated by crossing the *Prkar1a*^flox/+^ line (11) with the *Mlc2v-Cre*^+/−^ line (45) as described previously (9). *Prkar1a*^flox/+^/*Mlc2v-Cre*^−/−^ and *Prkar1a*^+/+^/*Mlc2v-Cre*^+/−^ littermates were used as controls. Both the *Prkar1a*^flox/+^ and the *Mlc2v-Cre*^+/−^ lines were backcrossed for at least eight generations to a C57BL/6 background. C57BL/6 mice and Sprague-Dawley rats were purchased from Envigo. All animal studies were approved by the Institutional Animal Care and Use Committee at Washington State University.

### Cell culture

NRCMs were isolated from 2- to 4-day-old Sprague-Dawley rats as described previously (9). In brief, hearts were digested with trypsin (50 μg/ml) and collagenase II (100 units/ml, Worthington Biochemical Corp.). NRCMs were cultured in serum-free medium 199 in 0.2% gelatin-coated plates. Cells were transfected with siRNAs using HiPerfect transfection reagent (Qiagen). The siRNA sequences used were as follows: *Prkar1a* siRNAs (siPRKAR1A), GACAGAUUCAGAGCCUACA[dT][dT]; p62 siRNAs (sip62), CAUGUCCUAUGUGAAAGAUGA[dT][dT]; and GFP siRNAs (siGFP), GGUGCGCUCCUGGACGUAGCC[dT][dT].

AMCMs were isolated as described previously (46), by enzymatic dissociation using collagenase II (0.5 mg/ml), collagenase IV (0.5 mg/ml), and protease XIV (0.05 mg/ml, Worthington Biochemical Corp.). AMCMs were cultured in serum-free medium 199 supplemented with bovine serum albumin (BP9706, Thermo Fisher Scientific, 0.1%), ITS (I3146, Sigma-Aldrich, 1%), BDM (B0753, Sigma-Aldrich, 10 mM), and CD lipid (11905-031, Thermo Fisher Scientific, 1%), in dishes coated with laminin (23017-15, Thermo Fisher Scientific, 5 μg/ml).

### Myocardial I/R surgery, infarct size, and cardiac function

Myocardial I/R surgery was performed in male and female mice (age 3–4 months) as described previously (47), by ligation of the left anterior descending (LAD) coronary artery for 30 min followed by reperfusion for indicated times. Infarct size was measured by Evans blue and 2,3,5-triphenyltetrazolium chloride (TTC) staining. In brief, at 24 h post I/R, LAD religation was performed and 2% Evans blue was systematically injected into mice to determine the nonischemic (blue) and ischemic (unstained, area at risk) tissue. The heart was then frozen at −80 °C for 10 min and cut into five slices. Heart slices were incubated in 2% TTC at 37 °C for 10 min to determine the viable (red) and infarcted (white) tissue. Infarct size was defined as the percentage of infarct area to area at risk or left ventricle. The areas were measured using ImageJ. Cardiac function was evaluated by echocardiography using VEVO 2100 (VisualSonics) under anesthesia with 1.5% isoflurane.

### Measurement of cardiomyocyte necrosis *in vivo*

Plasma membrane rupture, a major characteristic of necrosis that is not observed during apoptosis *in vivo*, was evaluated by Evans blue dye uptake assay as described previously (14). Briefly, mice received a single intraperitoneal injection of Evans blue (10 mg/ml, A16774, Alfa Aesar, 100 μg/g body weight) 16 h prior to I/R. At 24 h post I/R, mice were sacrificed and perfused retrogradely with 10 ml PBS. The heart was harvested and embedded in optimal cutting temperature (OCT) compound (Sakura), snap frozen in liquid nitrogen, and cut into 5-μm cryosections. Heart sections were stained with mouse anti-cardiac troponin T (cTnT, MS-295-P, Thermo Scientific, 1:100) to identify necrotic cardiomyocytes (Evans blue^+^/cTnT^+^). The percentage of necrotic cardiomyocytes was quantified with ImageJ.

### Measurement of cardiomyocyte necrosis *in vitro*

Necrosis *in vitro* was induced by stimulation of oxidative stress with H_2_O_2_ or calcium overload with ionomycin. Plasma membrane permeabilization was determined by PI exclusion and LDH release assays. In brief, cardiomyocytes were incubated with PI (P21493, Thermo Fisher, 2 μg/ml) for 30 min at 37 °C. Necrotic cells (PI^+^) were examined under a fluorescent microscope. LDH assay was performed using the Cytotoxicity Detection Kit (LDH) (11644793001, Roche) according to the manufacturer's instructions. In addition, cell viability was also assessed using Cell Proliferation Kit I (MTT, Roche). Both LDH and MTT assays were measured with Synergy NEO microplate reader (Biotek).

### Oxidative stress analysis

Oxidative stress was evaluated using 4-HNE *in vivo* and H2DCFDA *in vitro*. Hearts were fixed in 4% paraformaldehyde overnight and embedded in paraffin. Deparaffinized heart sections were subjected to antigen retrieval in 10 mmol/L citrate buffer (pH 6.0) and then incubated with rabbit anti-4-HNE (ab46545, Abcam, 1:100), mouse anti-cardiac Troponin T (MS-295-P, Thermo Scientific, 1:100), and DAPI (300 nM, D1306, Invitrogen). To analyze oxidative stress *in vitro*, cells were incubated with H2DCFDA (5 μM, D399, Invitrogen) at 37 °C for 30 min. H2DCFDA fluorescence intensity was measured with Synergy NEO microplate reader at Ex/Em: ∼492–495/517–527 nm (Biotek).

### Western blotting

Western blotting was performed using the following antibodies: rabbit anti-PKA R1α (ab139695, Abcam, 1:1000), rabbit anti-phospho-PKA substrate (9624, Cell Signaling, 1:1000), rat anti-Nrf2 (MABE1799, Millipore Sigma, 1:1000), rabbit anti-Nqo1 (ab34173, Abcam, 1:1000), mouse anti-p62 (ab56416, Abcam, 1:1000), rabbit anti-phospho-p62 (Ser349, ab211324, Abcam, 1:1000), rabbit anti-Ho-1 (82206, Cell Signaling, 1:1000), rabbit anti-Keap1 (7705, Cell Signaling, 1:1000), rabbit anti-LC3A/B (12741, Cell Signaling, 1:1000), rabbit anti-phospho-p70 S6 Kinase (Thr389, 9234, Cell Signaling, 1:1000), rabbit anti-p70 S6 Kinase (9202, Cell Signaling, 1:1000), rabbit anti-phospho-4E-BP1 (Thr37/46, 2855, Cell Signaling, 1:1000), rabbit anti-4E-BP1 (9644, Cell Signaling, 1:1000), rabbit anti-GAPDH (sc-25778, Santa Cruz Biotechnology, 1:1000), and mouse anti-β-actin (sc-47778, Santa Cruz Biotechnology, 1:1000).

### Quantitative RT-PCR

RT-PCR was performed using the iScript cDNA synthesis kit (1708840, Bio-Rad) and the iTaq Universal SYBR Green Supermix (1725121, Bio-Rad) with 18S rRNA as an internal control. Primer sequences were as follows: *Prkar1a*, 5′- CTGCTGAAGGACTCCATCGTG -3′ and 5′- ATCTGTCTTGCCTCCTCCTTCT -3′; *p62*/*Sqstm1*, 5′- GCACTACCGCGATGAGGAT-3′ and 5′- CCGGCACTCCTTCTTCTCTTTA -3′; *Nqo1*, 5′- AAGCTGCAGACCTGGTGAT-3′ and 5′- GGTCTTCTTATTCTGGAAAGGACC-3′; *Gstm1*, 5′-CAGAGTTCTTGAAGACCATCCCT -3′ and 5′- AATCCACATAGGTGACCTTGTCC-3′; *18S rRNA*, 5′-TGACTCAACACGGGAAACCTCAC-3′ and 5′-ATCGCTCCACCAACTAAGAACGG-3′.

### Statistical analysis

Statistical analysis was performed using Minitab and GraphPad Prism 7.02. Results are presented as mean ± SEM. Statistical differences between two groups were determined using two-tailed Student's *t* test. For multiple comparisons, one-way ANOVA followed by Dunnett's test or two-way ANOVA followed by Sidak test was used as appropriate. A value of *p* < 0.05 was considered statistically significant.

## Data availability

All data are contained within the article.

## Supporting information

This article contains supporting information.

## Conflict of interest

Dr Stratakis' laboratory at the NIH holds patents on *PRKAR1A* and related genes and/or their function and has received funding from Pfizer Inc. on research projects unrelated to the subject of this article.
