# Supplementary material for: Loss of PKA regulatory subunit 1α aggravates cardiomyocyte necrosis and myocardial ischemia/reperfusion injury
Source: J Biol Chem. 2021 Jun 1;297(1):100850. doi: 10.1016/j.jbc.2021.100850 (PMC8233231; doi:10.1016/j.jbc.2021.100850)
Supplement: Figures S1–S5 [file mmc1.pdf]

Loss of PKA regulatory subunit 1α aggravates cardiomyocyte necrosis and myocardial ischemia/reperfusion injury

Yuening Liu[1], Jingrui Chen[1], Peng Xia[1,*], Constantine A. Stratakis[2], Zhaokang Cheng[1,#]

From the [1] Department of Pharmaceutical Sciences, Washington State University, PBS 423, 412 E. Spokane Falls Blvd., Spokane, WA 99202-2131, USA; and the [2] Section on Endocrinology and Genetics, *Eunice Kennedy Shriver* National Institute of Child Health and Human Development, National Institutes of Health, 10 Center Drive, Building 10, NIH-Clinical Research Center, Room 1-3330, MSC1103, Bethesda, Maryland 20892, USA.
* Present address: Cardiovascular Research Center, Department of Medicine, Massachusetts General Hospital, Harvard Medical School, Boston, MA 02114, USA

Supporting Information

Supporting Information includes 5 Supplemental Figures with legends.

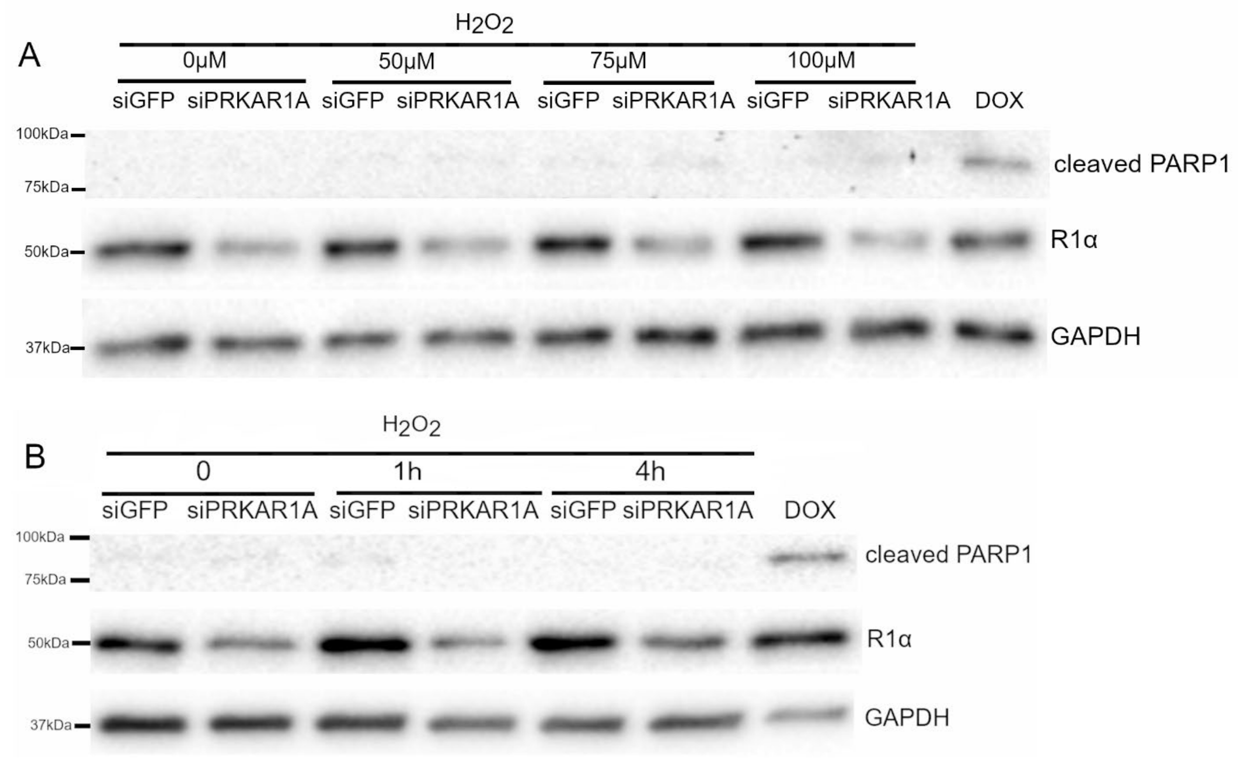

Figure S1. Disruption of *Prkar1a* gene (encoding R1α) did not potentiate cardiomyocyte apoptosis following treatment with H_2O_2. **(A)** NRCMs were transfected with control (siGFP) or *Prkar1a* siRNA (siPRKAR1A) for 48h prior to incubation with H_2O_2 at indicated concentrations for 4 h. NRCMs treated with doxorubicin (DOX, 1 μM) for 16 h served as a positive control for apoptosis. **(B)** NRCMs were transfected with siGFP or siPRKAR1A for 48h prior to incubation with H_2O_2 (100 μM) for indicated times. Protein levels were analyzed by western blotting. NRCMs treated with DOX (1 μM) for 16 h served as a positive control for apoptosis. Neither R1α loss nor H_2O_2 treatment increased the protein level of cleaved PARP1, an established marker of apoptosis.

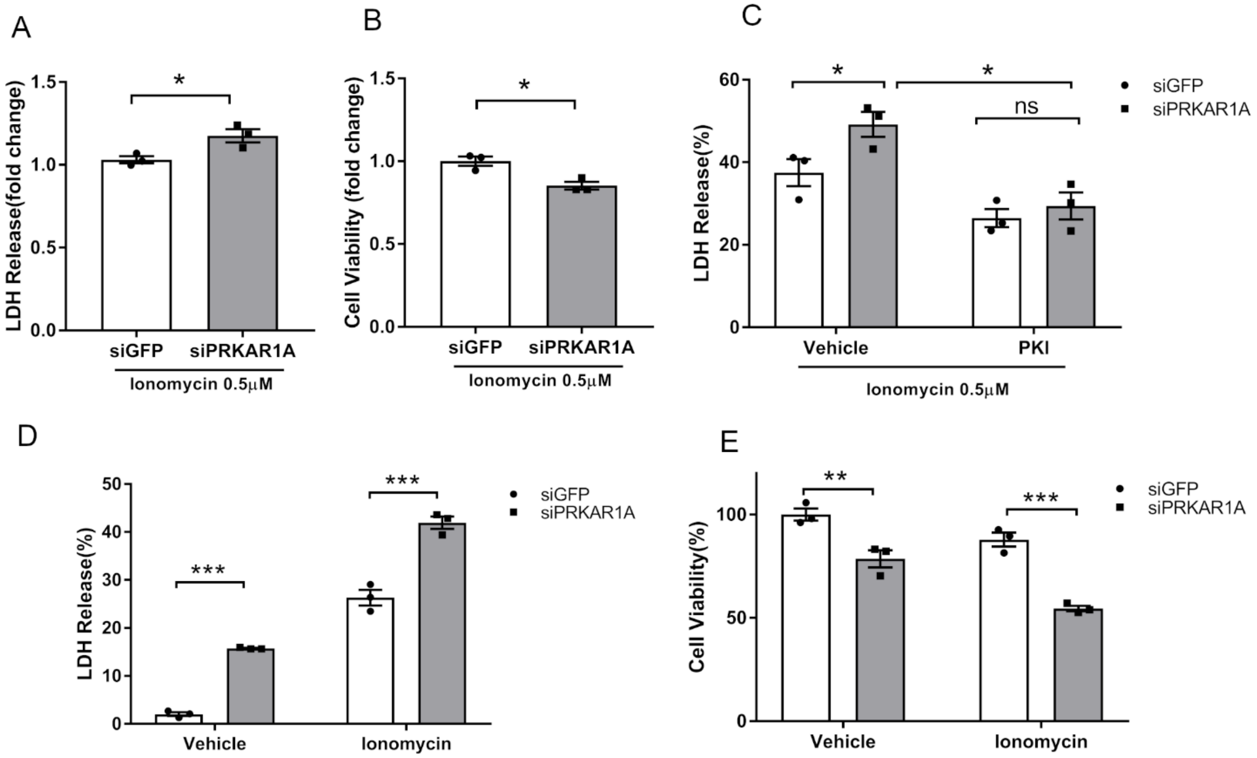

Figure S2. Knockdown of *Prkar1a* exacerbated ionomycin-induced cardiomyocyte necrosis through activation of PKA. **(A**, **B)** NRCMs were transfected with control (siGFP) or *Prkar1a* siRNA (siPRKAR1A) for 48h prior to incubation with ionomycin (0.5 μM) for 4 h. **(A)** Cardiomyocyte necrosis was determined by lactate dehydrogenase (LDH) release assay (n=3). Results are mean ± SEM and analyzed using two-tailed Student's *t* test. * $P<0.05$. **(B)** Cell viability was determined by MTT assay (n=3). Two-tailed Student's *t* test. * $P<0.05$. **(C)** NRCMs transfected with siGFP or siPRKAR1A were treated with ionomycin (0.5 μM) for 4 h, in the absence or presence of the small peptide PKA inhibitor (PKI, 5 μM). Cardiomyocyte necrosis was determined by LDH release assay (n=3). Two-way ANOVA with Sidak test. * $P<0.05$. ns, not significant. **(D**, **E)** H9c2 myoblasts were transfected with siGFP or siPRKAR1A for 48h prior to incubation with ionomycin (1 μM) for 4 h. **(D)** Cardiomyocyte necrosis was determined by LDH release assay (n=3). Two-way ANOVA with Sidak test. *** $P<0.001$. **(E)** Cell viability was determined by MTT assay (n=3). Two-way ANOVA with Sidak test. ** $P<0.01$, *** $P<0.001$.

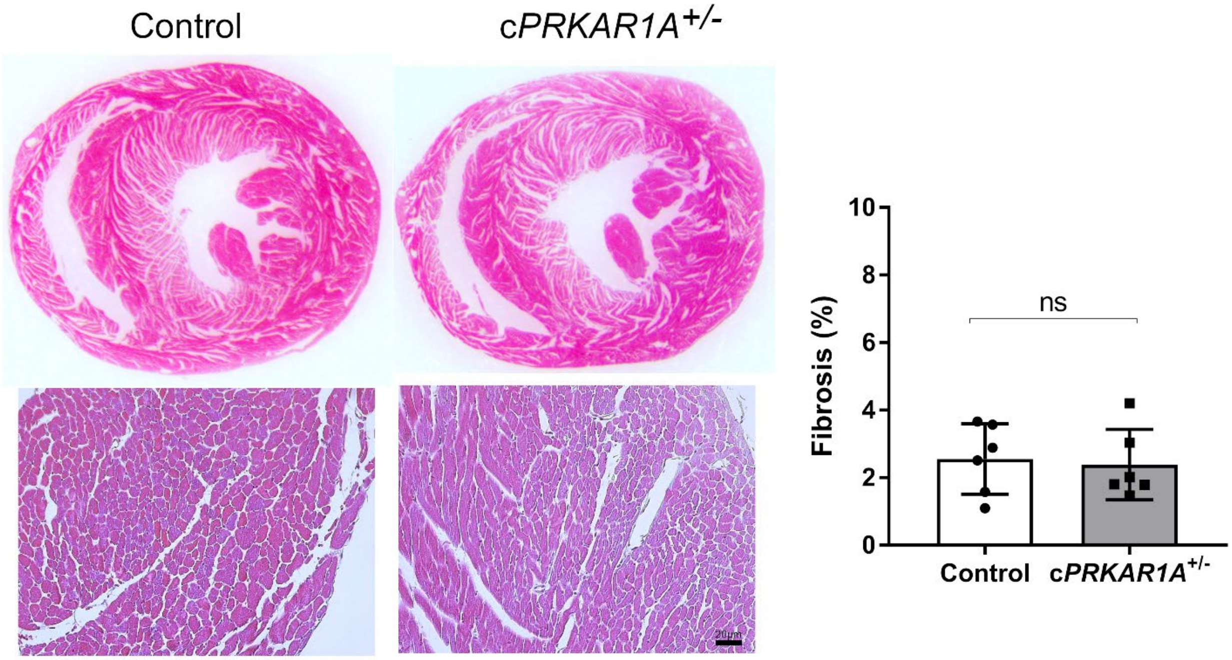

Figure S3. Myocardial fibrosis was minimal and comparable between control and c*PRKAR1A*$^{+/-}$ hearts at 3-4 months of age. Cardiac fibrosis in normal heart was evaluated by Masson's Trichrome staining (n=6 per group). Fibrosis was defined as the percentage of fibrotic area (*blue*) to left ventricle. Two-tailed Student's *t* test. ns, not significant. Scale bar=20 µm.

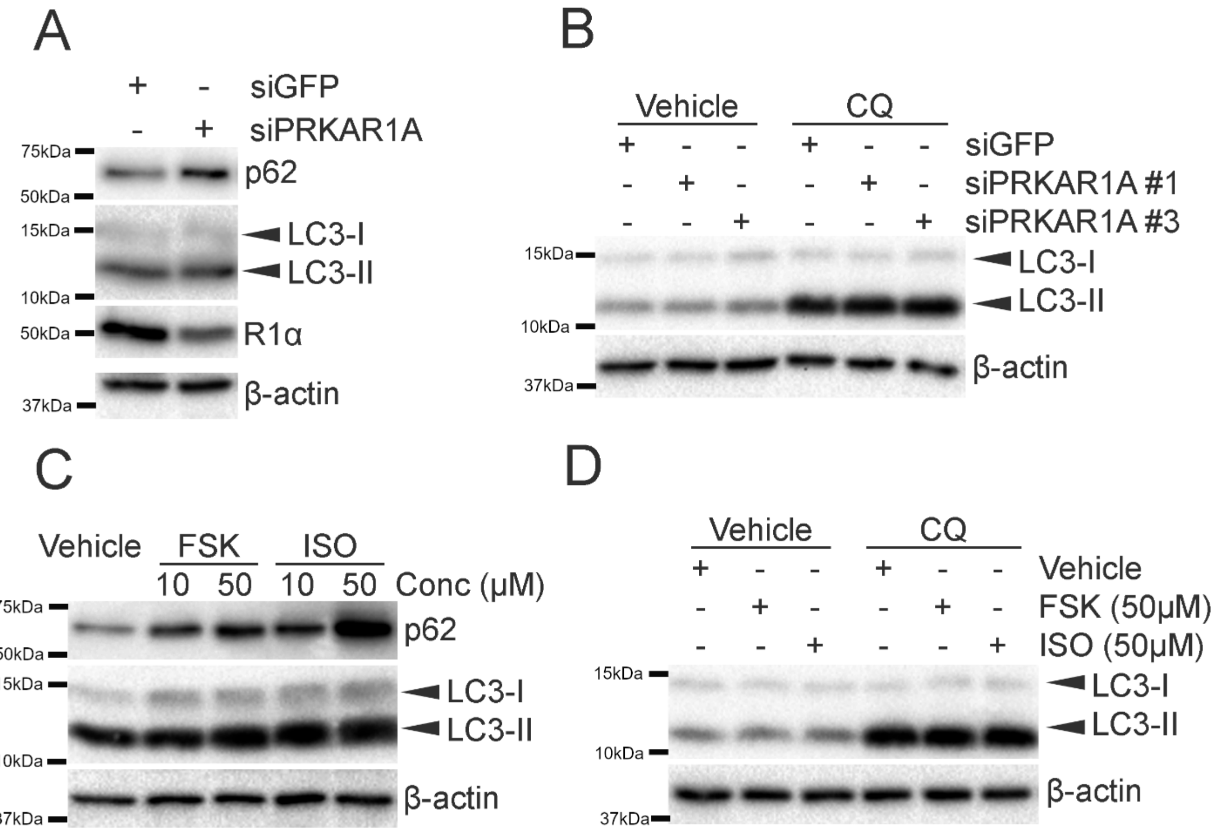

Figure S4. Disruption of R1α upregulated p62 without suppressing general autophagy. **(A)** NRCMs were transfected with control (siGFP) or *Prkar1a* siRNA (siPRKAR1A) for 48h. Protein levels were analyzed by western blotting. **(B)** NRCMs were transfected with siGFP or siPRKAR1A for 48h prior to incubation with the lysosome inhibitor chloroquine (CQ, 20μM) for 4 h. Protein levels were analyzed by western blotting. **(C)** NRCMs were treated with forskolin (FSK, 10 or 50 μM) or isoproterenol (ISO, 10 or 50 μM) for 24 h. Protein levels were analyzed by western blotting. **(D)** NRCMs were treated with FSK (50 μM) or ISO (50 μM) for 1 h prior to incubation with CQ (20 μM) for 4 h. Protein levels were analyzed by western blotting.

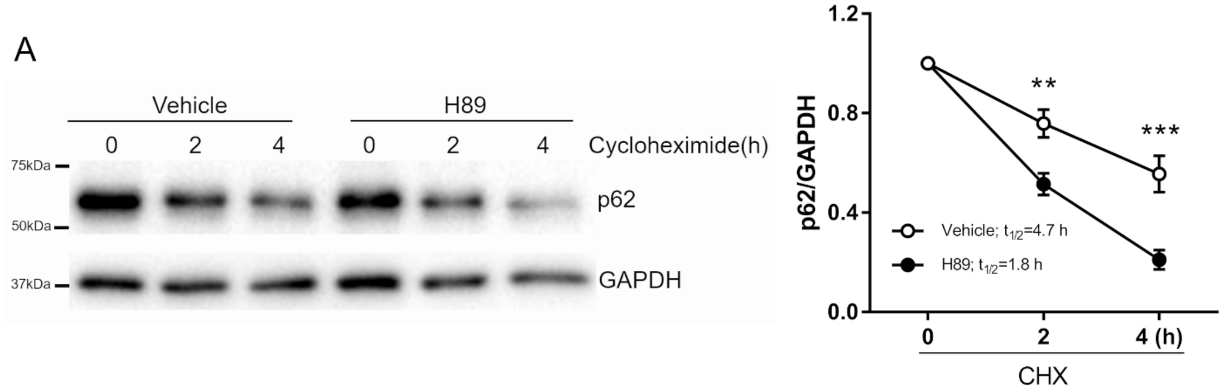

Figure S5. Treatment with the PKA inhibitor H89 enhanced p62 protein degradation. NRCMs were treated with H89 (10μM) for 4 h prior to incubation with the protein synthesis inhibitor cycloheximide (CHX, 10μg/ml) for various periods of time (n=3). Western blotting revealed that inhibition of PKA reduced the half-life of p62 protein. Two-way ANOVA with Sidak test. ** P<0.01, *** P<0.001 vs. Vehicle.
